# Association Between Menstrual Patterns and Adverse Pregnancy Outcomes in Patients With Polycystic Ovary Syndrome

**DOI:** 10.3389/fendo.2021.740377

**Published:** 2021-11-18

**Authors:** Ting Yu, Di Wu, Yurong Cao, Jun Zhai

**Affiliations:** ^1^ Center for Reproductive Medicine, The First Affiliated Hospital of Zhengzhou University, Zhengzhou, China; ^2^ Henan Key Laboratory of Reproduction and Genetics, The First Affiliated Hospital of Zhengzhou University, Zhengzhou, China; ^3^ Henan Provincial Obstetrical and Gynecological Diseases (Reproductive Medicine) Clinical Research Center, The First Affiliated Hospital of Zhengzhou University, Zhengzhou, China

**Keywords:** fresh IVF/ICSI embryo transfer, polycystic ovary syndrome, menstrual patterns, perinatal outcome, infertility

## Abstract

**Objective:**

To investigate the results of *in vitro* fertilization among polycystic ovary syndrome (PCOS) patients using the long-acting long protocol regarding the relationship between menstrual patterns and adverse pregnancy outcomes.

**Design:**

Retrospective cohort study.

**Setting:**

University-affiliated reproductive medical center.

**Background:**

The menstrual patterns of patients with PCOS is considered related to metabolism; however, no study has analyzed the outcome of *in vitro* fertilization/intracytoplasmic sperm injection (IVF/ICSI) in patients with PCOS who have different menstrual patterns. This study aimed to observe the outcomes of IVF/ICSI in patients with PCOS with different menstrual patterns who used the long-acting long protocol.

**Methods:**

This was a retrospective analysis in the first cycle of IVF/ICSI at the Reproductive Medicine Center of the First Affiliated Hospital of Zhengzhou University from January 2017 to December 2019. In total, 1834 patients with PCOS were classified into the regular menstruation group (n=214), the oligomenorrhea group (n=1402), and the amenorrhea group (n=218).

**Results:**

PCOS patients who used the long-acting long protocol of IVF/ICSI had similar clinical pregnancy rates and live birth rates despite having different menstrual patterns. The overall incidence of adverse pregnancy outcomes, including abortion, spontaneous preterm birth (sPTB), gestational diabetes(GDM), hypertensive disorder inpregnancy (HDP), and premature rupture of membranes(PROM, was significantly higher in the amenorrhea group than in the regular menstrual and oligomenorrhea groups (25.88% *vs*. 30.41% *vs*. 43.69%; P = 0.013). Additionally, the rates of GDM (2.35% *vs*. 6.10% *vs*. 13.79%; P=0.015) and macrosomia (5.26% *vs*. 10.94% *vs*. 18.39%; P=0.026) in the amenorrhea group were significantly higher than those in the other two groups. Correction for confounding factors showed that menstrual patterns are related to the occurrence of adverse pregnancy outcomes. Amenorrhea is an independent risk factor for adverse pregnancy outcome (OR [odds ratio]: 2.039, 95% CI [confidence interval]: 1.087-3.822), GDM (OR: 5.023, 95% CI: 1.083–23.289), and macrosomia (OR: 4.918, 95% CI: 1.516–15.954).

**Conclusions:**

IVF/ICSI can achieve similar pregnancy and live birth rates in PCOS patients with different menstrual patterns. However, the overall incidence of adverse pregnancy outcomes in PCOS patients with amenorrhea is higher than that in patients with regular menstruation or oligomenorrhea.

## 1 Introduction

Polycystic ovary syndrome (PCOS) is a common reproductive and endocrine disease among women of childbearing age. It is characterized by abnormal ovulation (sparse ovulation or anovulation), polycystic ovarian morphology (PCOM), and hyperandrogenism (HA) (1). Studies have shown that the incidences of abortions, premature births, and pregnancy complications among PCOS patients are relatively high. Therefore, it is necessary to strengthen clinical perinatal monitoring (2). Currently, conclusions on related risk factors that lead to adverse pregnancy outcomes in patients with PCOS are not completely consistent. Some of these risk factors include age, body mass index (BMI), and the application of assisted reproductive technology (3). Patients with PCOS have heterogeneous clinical presentations. The most common menstrual manifestation is oligomenorrhea or amenorrhea, and there are differences in the levels of sex hormones and metabolic factors among patients with different menstrual patterns (4). Studies have shown that menstrual disorders in patients with PCOS are caused by insulin resistance (IR) and HA, which indicates that menstrual patterns may be used as a direct clinical observation index of the severity of metabolic disorders (5). Currently, there is no published research on the outcomes of *in vitro* fertilization (IVF)/intracytoplasmic sperm injection (ICSI) in patients with PCOS that have different menstrual patterns, and it is unclear whether menstrual patterns affect the pregnancy outcomes of patients. In recent years, due to the improvements in endometrial receptivity and the pregnancy rate of fresh cycle embryo transfer, the long-acting long protocol has been applied at some reproductive medicine centers in China (6). This study retrospectively analyzed the pregnancy outcomes of patients with PCOS that have different menstrual patterns during the long-acting long protocol assisted by IVF/ICSI and the related risk factors for adverse pregnancy outcomes. This analysis further aimed to enable clinicians to strengthen the treatment of high-risk groups, as perinatal monitoring can reduce the incidence of maternal and infant adverse outcomes.

## 2 Materials and Methods

### 2.1 Study Design and Patients

**Figure 1:**
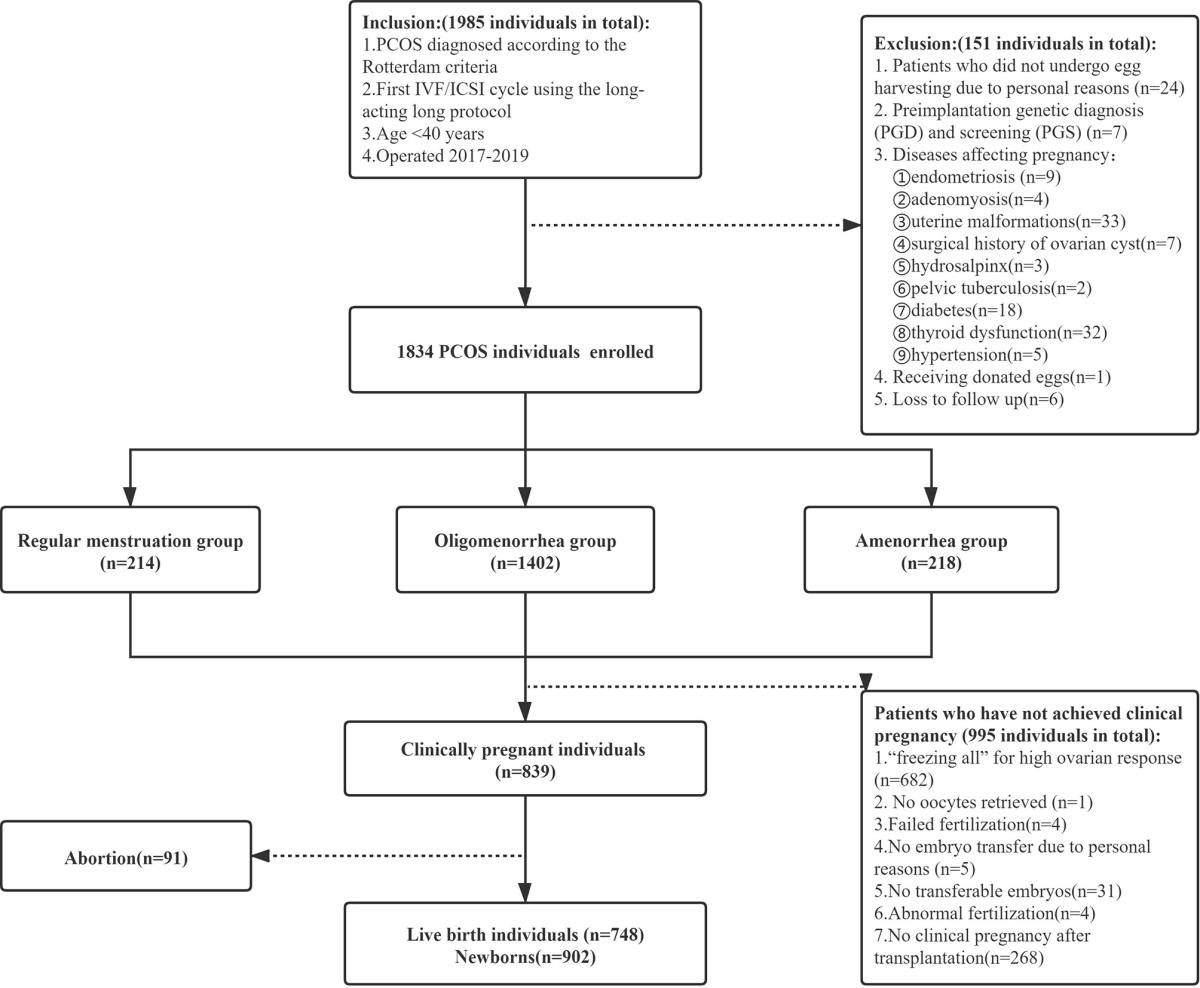
Flow chart depicting the patient selection.

This retrospective cohort study was approved by the Ethics Review Committee of the First Affiliated Hospital of Zhengzhou University. Written informed consent was waived due to the retrospective nature of the study. The selected patients underwent IVF/ICSI therapy at the Reproductive Medicine Center of the First Affiliated Hospital of Zhengzhou University from January 2017 to December 2019. The inclusion criteria were as follows: (1) receiving IVF/ICSI in the first cycle (the patient underwent IVF/ICSI because of the diagnosis of infertility, male factors or AIH failure, et al.), (2) undergoing the long-acting long protocol, (3) age <40 years, (4) Diagnosis of PCOS is based on 2 of 3 Rotterdam Criteria: a) Hyperandrogenism – either clinically by skin manifestations of androgen excess OR hyperandrogenemia (high testosterone in a blood test); b) Ovulation dysfunction (i.e. Oligo/Anovulation); c) Polycystic ovaries on ultrasound. Exclusion of phenotypically similar androgen excess disorders such as congenital adrenal hyperplasia (CAH), androgen-secreting tumors, Cushing syndrome, thyroid dysfunction, and hyperprolactinemia (7). In contrast, the exclusion criteria were as follows: (1) patients who did not undergo egg harvesting due to personal reasons; (2) Preimplantation genetic diagnosis (PGD) and screening (PGS); (3) Diseases affecting pregnancy: endometriosis, adenomyosis, uterine malformations, surgical history of ovarian cyst, hydrosalpinx, pelvic tuberculosis, diabetes, thyroid dysfunction, hypertension; (4) receipt of donor eggs; and (5) Loss to follow up. We classified the patients with PCOS according to menstrual patterns as documented in the new classification of the causes of abnormal uterine bleeding in the non-pregnant women of childbearing age (PALM-COEIN) system published by the International Federation of Gynecology and Obstetrics in 2011 (8). The patients with PCOS were classified into the regular menstruation group (21 days ≤ the menstrual cycle ≤ 35 days), the oligomenorrhea group (35 days < menstrual cycle ≤180 days), and amenorrhea group (menstrual cycle >180 days) (**Figure 1**).

### 2.2 IVF/ICSI PROTOCOLS

#### 2.2.1 Downregulation Regimen

On the second to third days of the menstrual cycle, 3.75 mg of a long-acting gonadotropin-releasing hormone agonist (GnRH-a; The Ipsen Group, Dauphine, France) was administered to achieve downregulation. After 30–42 days, the downregulating standard was reached (no ovarian cyst with diameter >10 mm, an estradiol level of <183 pmol/L, and a luteinizing hormone [LH] level <3 IU/L), and controlled ovarian hyperstimulation was performed.

#### 2.2.2 Ovulation Induction

Ovarian induction therapy was administered according to BMI, and the level of anti-Müllerian hormone (AMH) was used to determine the dosage of gonadotropins (Gonal-f; Merck Serono, Switzerland or Plycon; Organon, Netherlands). According to follicle size and hormone levels, we adjusted the dosage of gonadotropins and considered the addition of human menopausal gonadotropin (LeBold, Zhuhai Livzon Pharmaceutical, China).

#### 2.2.3 HCG Injection and Corpus Luteum Support Standard

We administered 250 μg of Azer (Merck Serono, Italy) and 2000 IU of human chorionic gonadotropin (hCG) (Zhuhai Livzon Pharmaceutical) when the diameter of one dominant follicle was ≥20 mm, the diameters of three follicles were ≥17 mm, or two-thirds of the follicles had diameters ≥16 mm. The eggs were harvested under vaginal ultrasound guidance 36–37 h after the injections were administered.

#### 2.2.4 IVF/ICSI-ET

The method of fertilization was based on semen quality. Fresh embryo transfer was performed 3–5 days after oocyte retrieval based on embryo quality and the endometrial and patient’s conditions. The transplant was cancelled if patients were deemed to be at high risk for OHSS, the P level was >3 ng/mL, or a uterine effusion was demonstrated. Depending on the specific situation of each patient, different frozen-thawed ET protocols were formulated.

### 2.3 Follow-Up of Patients

In terms of following up pregnancy outcomes, 14 days after embryo transfer, blood was drawn for β-hCG determination, and follow-up was continued until 35 days after transplantation. Clinical pregnancy was diagnosed when an ultrasonic examination revealed a gestational sac. Fetal nuchal translucency examination was performed at 12 weeks of pregnancy (9–10 weeks after embryo transfer), and prenatal care was provided at the department of obstetrics. During the perinatal period, trained nurses provided follow-up *via* telephone. Standardized questionnaires were used to collect information on perinatal complications, gestational age, birth date, mode of delivery, neonatal sex and birth weight, diseases among newborns, treatment, and prognosis. The follow-up information was recorded in detail and stored in the electronic medical records together with the previous treatment information. The research data were extracted from the electronic database of our hospital.

### 2.4 Clinical Outcomes

Abortion: the spontaneous loss of a clinical pregnancy that occurs before 20 completed weeks of gestational age (18 weeks post fertilization) (9). Spontaneous preterm birth (sPTB): A delivery with spontaneous onset before 37 weeks of gestation (10). Gestational diabetes (GDM): Defined as abnormal glucose metabolism during pregnancy, without a pre-pregnancy diagnosis of diabetes (11). Hypertensive disorder in pregnancy (HDP): Either Pregnancy-induced hypertension (PIH): the new onset of hypertension (≥ 140 mmHg systolic and/or ≥90 mmHg diastolic blood pressure) after 20 weeks gestation measured on at least two occasions four hours apart; Pre-eclampsia (PE): PIH accompanied by proteinuria (≥300 mg in 24 hours), either during pregnancy or postpartum (12, 13). Premature rupture of membranes (PROM): Premature rupture of membranes (PROM) is a rupture (breaking open) of the membranes (amniotic sac) before labor begins. Adverse pregnancy outcomes: women with one of the following complications, including abortion, sPTB, GDM, HDP, and PROM (14). Macrosomia: Birth weight more than 4,000 grams. Low birth weight(LBW): Birth weight less than 2,500 grams. Very low birth weight (VLBW): Birth weight less than 1,500 grams. Implantation rate: the number of gestational sacs observed, divided by the number of embryos transferred. Clinical pregnancy rate: the number of clinical pregnancies, divided by the number of transplant cycles. Live birth rate: the number of deliveries that resulted in at least one live born baby, divided by the number of transplant cycles (9). Adverse pregnancy outcomes rate: the number of women with adverse pregnancy outcomes, divided by the numbers of clinical pregnancies. Macrosomia rate: the numbers of deliveries with at least one macrosomia Infant, divided by the numbers of deliveries. LBW rate: the numbers of deliveries with at least one LBW Infant, divided by the numbers of deliveries. VLBW rate: the numbers of deliveries with at least one VLBW Infant, divided by the numbers of deliveries (15, 16).

### 2.5 Statistical Analysis

To compare continuous variables between multiple groups, a one-way ANOVA test was used. Data with normal distributions are reported as means and standard deviations. The LSD-t-test was used for pairwise comparison of continuous variables within the group, Fisher’s exact tests or chi-square test was used to compare proportions (Bonferroni correction was used to account for multiple testing).Variables that were statistically significant in the univariate t-tests and chi-squared tests were included in the multivariate logistic regression analysis. The results are reported as adjusted odds ratios (aORs) with 95% confidence intervals (CIs). All statistical analyses were performed using SPSS version 24.0 (IBM Corporation, Armonk, NY, USA). Analysis items with p-values of <0.050 were considered statistically significant.

## 3 Results

### 3.1 Comparison of Patients’ General Characteristics and Ovulation Induction Information

In total, 1834 patients were recruited in this study, including 214 in the regular menstrual group, 1402 in the oligomenorrhea group, and 218 in the amenorrhea group. The length of infertility, BMI, LH/FSH, AMH, AFC, Length of stimulation, and Total dosage of Gn used were higher, and the thickness on the hCG injection day was lower in the amenorrhea group than in the regular menstruation and oligomenorrhea groups. There were differences between groups (P<0.017). There was no statistical difference in the other indicators (**Table 1**).

**Table 1:** Comparison of patients’ general characteristics and ovulation induction information.

Item	Regular menstruation group (n = 214)	Oligomenorrhea group (n = 1402)	Amenorrhea group (n = 218)	P value
Age (year)	28.71 ± 3.83	28.61 ± 3.54	28.84 ± 3.68	0.635
Type of infertility				0.115
Primary infertility (%)	65.89 (141/214)	65.3 (916/1402)	72.48 (158/218)	
Secondary infertility (%)	34.11 (73/214)	34.66 (486/1402)	27.52 (60/218)	
Duration of infertility (year)	3.8 ± 2.62	3.82 ± 2.53^a^	4.31 ± 2.80	0.035
BMI (kg/m2)	24.46 ± 3.46	24.16 ± 3.31^a^	24.75 ± 3.25	0.035
E2 (pg/ml)	41.35 ± 27.00	50.61 ± 56.52	53.38 ± 49.09	0.036
P (ng/ml)	0.47 ± 0.33	0.76 ± 1.86	0.75 ± 1.83	0.088
T (ng/ml)	0.78 ± 3.72	0.87 ± 5.19	0.70 ± 3.63	0.883
LH/FSH (mIU/ml)	1.54 ± 1.06^AB^	1.75 ± 1.26^a^	2.19 ± 1.24	<0.001
AMH (ng/ml)	7.93 ± 3.63^B^	7.93 ± 3.95^a^	9.85 ± 4.87	<0.001
AFC (n)	23.83 ± 0.58^AB^	22.26 ± 4.83	22.52 ± 4.43	<0.001
Starting dose of Gn (IU)	107.77 ± 18.18	107.61 ± 18.03	108.20 ± 18.18	0.903
Length of stimulation (d)	14.56 ± 2.57^B^	14.48 ± 2.61^a^	15.13 ± 2.78	0.003
Total dosage of Gn used (IU)	2157.36 ± 852.28	2117.23 ± 842.65^a^	2308.54 ± 894.45	0.008
hCG injection day				
Endometrial thickness (mm)	12.49 ± 2.54^B^	12.43 ± 2.43^a^	11.76 ± 2.16	0.001
E2 (pg/ml)	4120.67 ± 2387.82	4022.39 ± 2261.54	4060.75 ± 2013.99	0.828
LH (mIU/ml)	0.76 ± 0.86	0.72 ± 0.94	0.63 ± 0.65	0.285
P (ng/ml)	0.92 ± 0.58	0.83 ± 0.54	0.81 ± 0.53	0.080

Continuous data: mean ± SD. Categorical data: % (n/N); BMI, body mass index; E2, estradiol; P, progesterone; T, testerone; LH, luteinizing hormone; FSH, follicle-stimulating hormone; AMH, Anti-Müllerian Hormone; AFC, Antral follicular count; Gn, gonadotropin; hCG, human chorionic gonadotropin.

^A^P, ^B^P indicate Regular menstruation group vs. Oligomenorrhea group and Amenorrhea group respectively. ^a^P indicate Oligomenorrhea group vs. Amenorrhea group.

### 3.2 Laboratory Results and Clinical Outcomes

**Figure 2:**
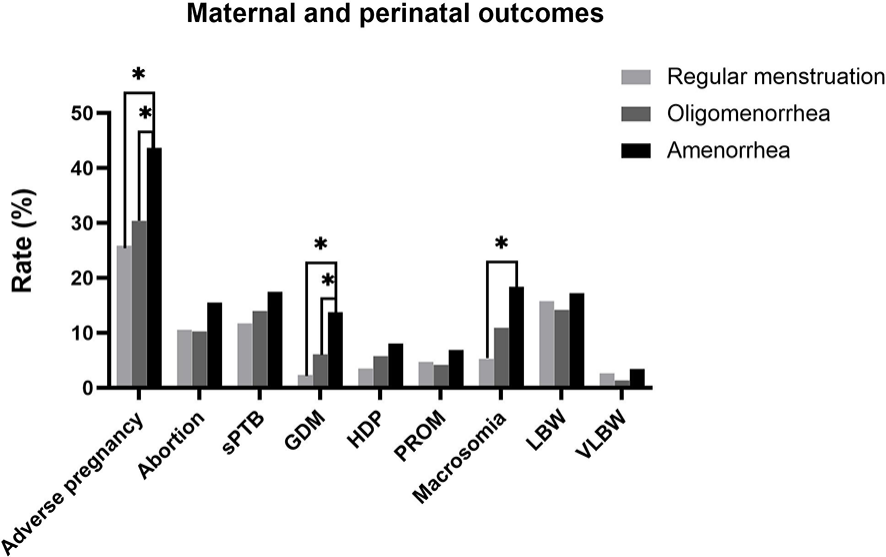
Maternal and perinatal outcomes.*: Bonferroni correction, P < 0.050.

The overall incidence of adverse pregnancy outcomes(abortion, sPTB, GDM, HDP, and PROM) was significantly higher in the amenorrhea group than in the regular menstruation and oligomenorrhea groups (25.88% vs. 30.41% vs. 43.69%; P=0.013). Further, the rate of GDM (2.35% vs. 6.10% vs. 13.79%; P=0.015) and macrosomia (5.26% vs. 10.94% vs. 18.39%; P=0.026) were higher in the amenorrhea group than in the other two groups. Abortion, sPTB, HDP, PROM, and the incidence of LBW and VLBW had a tendency to increase in the amenorrhea group compared with the regular menstruation group and the oligomenorrhea group; however, there was no significant difference (**Table 2** and **Figure 2**).

**Table 2:** Laboratory results and clinical outcomes.

	Regular menstruation group (n = 214)	Oligomenorrhea group (n = 1402)	Amenorrhea group (n = 218)	P value
No. of oocytes retrieved (n)	19.22 ± 7.83	18.47 ± 7.69	18.69 ± 7.58	0.561
Fertilization method				0.195
IVF	89.72 (192/214)	87.09 (1221/1402)	90.83 (198/218)	
ICSI	10.28 (22/214)	12.91 (181/1402)	9.17 (20/218)	
No. of 2PN (n)	11.72 ± 6.00	11.23 ± 5.52	11.14 ± 5.57	0.741
No. of 2PN cleavage (n)	11.58 ± 5.98	11.09 ± 5.47	11.02 ± 5.53	0.724
No. of high-quality embryo (n)	6.92 ± 4.58	6.90 ± 4.46	6.95 ± 4.52	0.519
Total 2PN fertilization rate (%)	60.09 (2472/4114)	59.66 (15448/25892)	59.34 (2418/4075)	0.785
IVF 2PN fertilization rate (%)	62.58 (2233/3568)	60.87 (13376/21973)	60.27 (2178/3614)	0.094
ICSI 2PN fertilization rate (%)	58.87 (239/406)	60.94 (2072/3400)	60.61 (240/396)	0.720
Cleavage rate (%)	98.87 (2444/2472)	98.78 (15260/15448)	98.92 (2392/2418)	0.115
Abnormal fertilization rate (%)	0.47 (1/214)	0.14 (2/1402)	0.46 (1/218)	0.459
No transferable embryos rate (%)	2.34 (5/214)	1.57 (22/1402)	1.83 (4/218)	0.709
“freezing all” for high ovarian response (%)	35.05 (75/214)	32.10 (450/1402)	33.03 (72/218)	0.683
Implantation rate (%)	52.22 (106/203)	60.15 (821/1365)	62.44 (133/213)	0.065
Biochemical pregnancy rate (%)	70.63 (89/126)	80.40 (681/847)	80.60 (108/134)	0.038
Clinical pregnancy rate (%)	67.46 (85/126)	76.86 (651/847)	76.86 (103/134)	0.068
Adverse pregnancy rate (%)	25.88 (22/85)^B^	30.41 (198/651)^a^	43.69 (45/103)	0.013
Abortion rate (%)	10.58 (9/85)	10.29 (66/651)	15.53 (16/103)	0.263
sPTB rate (%)	11.74 (12/85)	13.98 (91/651)	17.48 (18/103)	0.510
GDM rate (%)	2.35 (2/85)^B^	6.10 (35/651)^a^	13.79 (12/103)	0.015
HDP rate (%)	3.53 (3/85)	5.75 (33/651)	8.05 (7/103)	0.581
PROM (%)	4.71 (4/85)	4.18 (24/651)	6.90 (6/103)	0.565
Live birth rate (%)	60.32 (76/126)	69.07 (585/847)	64.93 (87/134)	0.115
Gestational week of childbirth (d)	37.54 ± 2.30	37.83 ± 2.06	37.45 ± 2.67	0.195
No. of live babies delivered				0.063
Singletons (%)	89.47 (68/76)	77.95 (456/585)	80.46 (70/87)	
Multiples (%)	10.53 (8/76)	22.05 (129/585)	19.54 (17/87)	
Macrosomia rate (%)	5.26 (4/76)^B^	10.94 (64/585)	18.39 (16/87)	0.026
LBW rate (%)	15.79 (12/76)	14.19 (83/585)	17.24 (15/87)	0.725
VLBW rate (%)	2.63 (2/76)	1.37 (8/585)	3.45 (3/87)	0.295

Continuous data: mean ± SD. Categorical data: % (n/N); 2PN, 2 pronuclei; OHSS, ovarian hyperstimulation syndrome; adverse pregnancy (including abortions, sPTB, GDM, HDP, and PROM); sPTB, spontaneous preterm birth; GDM, gestational diabetes; HDP, hypertensive disorder in pregnancy; PROM, premature rupture of membranes; Adverse pregnancy (including abortions, sPTB, GDM, HDP, and PROM); LBW,Infant low birth weight; VLBW, Infant very low birth.

^A^P, ^B^P indicate Regular menstruation group vs. Oligomenorrhea group and Amenorrhea group respectively. ^a^P indicate Oligomenorrhea group vs. Amenorrhea group.

### 3.3 Logistic Regression Assessment of Perinatal Outcomes

**Figure 3:**
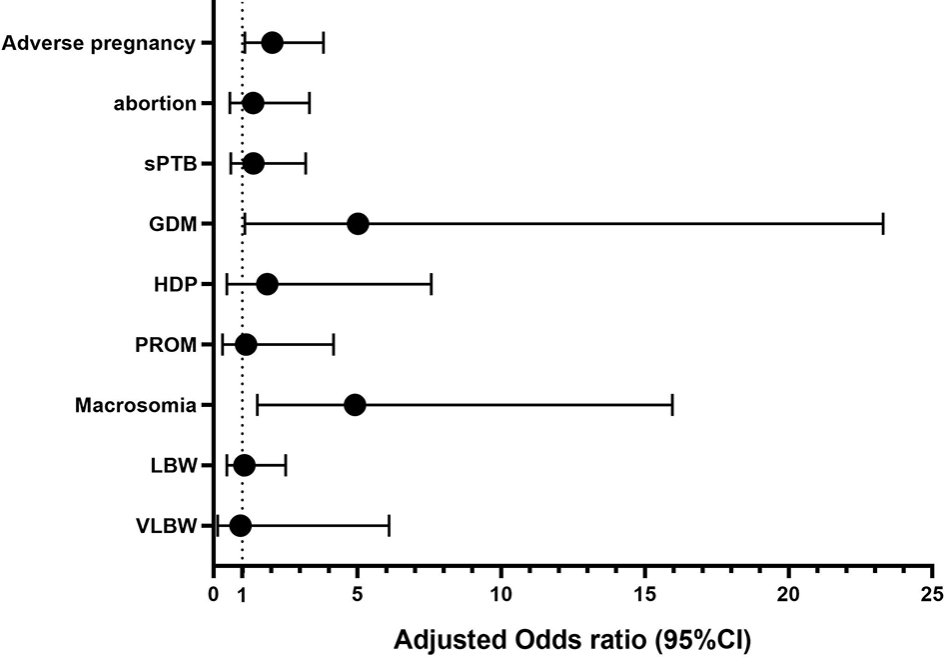
Adjusted OR (95%CI) of Amenorrhea and maternal and perinatal outcomes. Adverse pregnancy adjusted by BMI and hCG injection day endometrial thickness; GDM adjusted by Age and BMI; Abortion adjusted by LH/FSH and AMH; Macrosomia adjusted by BMI, AFC, Starting dose of Gn, No. of oocytes retrieved and Multiples rate; sPTB adjusted by AFC and hCG injection day endometrial thickness; HDP adjusted by BMI, Starting dose of Gn, hCG injection day E2 and LH; PROM adjusted by hCG injection day endometrial thickness; LBW adjusted by AFC, Length of stimulation, Total dosage of Gn used, hCG injection day Endometrial thickness, No. of oocytes retrieved and Multiples rate; VLBW adjusted by Multiples rate.

In total, 839 patients achieved clinical pregnancies, and univariate logistic analysis of adverse pregnancy outcomes (abortion, sPTB, GDM, HDP, and PROM) and other outcome indicators was performed. Compared with the regular menstruation group, amenorrhea was the risk factors of adverse pregnancy outcome (OR [odds ratio]: 2.222, 95% CI [confidence interval]: 1.193–4.139), GDM (OR: 5.473, 95% CI: 1.189–25.178), and macrosomia (OR: 4.056, 95% CI: 1.293–12.729). The indicators with statistical differences in menstrual patterns in the univariate analysis were included in the multivariate logistic analysis. After correction for confounding factors, amenorrhea was revealed as independent risk factors for adverse pregnancy outcomes (OR: 2.039, 95% CI: 1.087–3.822), GDM (OR: 5.023, 95% CI: 1.083–23.289), and macrosomia (OR: 4.918, 95% CI: 1.516–15.954). Additionally, BMI was also an independent risk factor for adverse pregnancy outcomes (OR: 1.046, 95% CI: 1.001–1.093), GDM (OR: 1.117, 95% CI: 1.022–1.22), and macrosomia (OR: 1.021, 95% CI: 1.004–1.037). The menstrual pattern is not an independent influencing factor of abortion, sPTB, PROM, LBW, and VLBW (P>0.050). (**Table 3** and **Figure 3** and **Supplementary Tables 1–6** in Supplementary Materials).

**Table 3:** Logistic regression analysis of maternal and perinatal outcomes.

	menstrual patterns	OR (95%CI)	P	OR^a^ (95%CI)	P^a^
Adverse pregnancy			0.014		0.042
	regular menstruation	Reference	–	Reference	–
	oligomenorrhea	1.252 (0.749,2.091)	0.391	1.246 (0.742,2.091)	0.405
	amenorrhea	2.222 (1.193,4.139)	0.012	2.039 (1.087,3.822)	0.026
Abortion			0.267		0.439
	regular menstruation	Reference	–	Reference	–
	oligomenorrhea	0.953 (0.456,1.989)	0.323	0.924 (0.44,1.937)	0.834
	amenorrhea	1.553 (0.649,3.717)	0.897	1.374 (0.566,3.332)	0.483
sPTB			0.512		0.675
	regular menstruation	Reference	–	Reference	–
	oligomenorrhea	1.219 (0.608,2.445)	0.276	1.096 (0.542,2.22)	0.798
	amenorrhea	1.588 (0.69,3.653)	0.577	1.382 (0.596,3.203)	0.451
GDM			0.020		0.048
	regular menstruation	Reference	–	Reference	–
	oligomenorrhea	2.358 (0.557,9.985)	0.244	2.462 (0.579,10.47)	0.222
	amenorrhea	5.473 (1.189,25.178)	0.029	5.023 (1.083,23.289)	0.039
HDP			0.599		0.663
	regular menstruation	Reference	–	Reference	–
	oligomenorrhea	1.46 (0.438,4.866)	0.538	1.392 (0.411,4.71)	0.595
	amenorrhea	1.993 (0.499,7.956)	0.329	1.862 (0.458,7.567)	0.385
PROM			0.568		0.648
	regular menstruation	Reference	–	Reference	–
	oligomenorrhea	0.775 (0.262,2.29)	0.645	0.754 (0.254,2.239)	0.612
	amenorrhea	1.253 (0.342,4.592)	0.734	1.129 (0.306,4.167)	0.855
Macrosomia			0.032		0.028
	regular menstruation	Reference	–	Reference	–
	oligomenorrhea	2.211 (0.782,6.254)	0.135	2.992 (1.036,8.647)	0.043
	amenorrhea	4.056 (1.293,12.729)	0.016	4.918 (1.516,15.954)	0.008
LBW			0.726		0.529
	regular menstruation	Reference	–	Reference	–
	oligomenorrhea	0.882 (0.456,1.704)	0.708	0.787 (0.400,1.548)	0.488
	amenorrhea	1.111 (0.484,2.549)	0.804	1.071 (0.457,2.507)	0.875
VLBW			0.336		0.186
	regular menstruation	Reference	–	Reference	–
	oligomenorrhea	0.513 (0.107,2.462)	0.404	0.322 (0.063,1.645)	0.173
	amenorrhea	1.321 (0.215,8.125)	0.764	0.934 (0.143,6.105)	0.943

Reference, This variable functions as an indicator; ^a^OR, ^a^P, adjusted by the variables which were statistically significant in the univariate logistic analysis; Adverse pregnancy adjusted by BMI and hCG injection day endometrial thickness; GDM adjusted by Age and BMI; Abortion adjusted by LH/FSH and AMH; Macrosomia adjusted by BMI, AFC, Starting dose of Gn, No. of oocytes retrieved and Multiples rate; sPTB adjusted by AFC and hCG injection day endometrial thickness; HDP adjusted by BMI, Starting dose of Gn, hCG injection day E2 and LH; PROM adjusted by hCG injection day endometrial thickness; LBW adjusted by AFC, Length of stimulation, Total dosage of Gn used, hCG injection day Endometrial thickness, No. of oocytes retrieved and Multiples rate; VLBW adjusted by Multiples rate.

## 4 Discussion

This study analyzed the relationship between menstrual patterns and pregnancy outcomes in PCOS patients undergoing IVF/ICSI for the first cycle. The results show that the menstrual pattern of PCOS patients is related to adverse pregnancy outcomes (abortion, sPTB, GDM, HDP, and PROM). Amenorrhea is an independent risk factors for adverse pregnancy outcomes, GDM and macrosomia. In addition, menstrual patterns do not affect the clinical pregnancy and live birth rates of IVF/ICSI-assisted pregnancy in PCOS patients.

According to the Rotterdam criteria, PCOS patients experience oligomenorrhea or amenorrhea; however, some patients have regular menstruation. Presently, the cause of PCOS is unclear, and hyperandrogenism and hyperinsulinemia are its pathophysiological characteristics (17, 18). Hypothalamic pituitary dysfunction leads to ovulation disorders in PCOS patients and causes menstrual irregular and infertility (19). Hormonal and metabolic abnormalities in PCOS patients may increase the risk of obstetric and neonatal complications (20). Many studies have shown that PCOS is related to adverse perinatal outcomes, such as GDM (21), PROM (22), and HDP (23). In addition, PCOS in pregnancy is similarly associated with adverse neonatal outcomes and an increase in the incidences of premature delivery, intrauterine growth restriction, abnormal birth weight (smaller or greater than gestational age infants), and admission to the neonatal intensive care unit (24).

Some recent studies have suggested that adverse pregnancy outcomes in PCOS patients may be related to menstrual patterns. A prospective cohort study showed that a HA + menstrual dysfunction + PCO phenotype is a risk factor for adverse pregnancy and neonatal outcomes, including gestational diabetes, pre-eclampsia, and reduced weight babies, in PCOS patients (25, 26). A study of PCOS patients with normal menstrual cycle or oligomenorrhea and natural pregnancies showed that the risk of pregnancy complications in patients with PCOS may be affected by menstrual patterns, and the risk of pregnancy complications in patients with normal ovulation was similar to that of the normal population; however, no analysis was conducted among patients with PCOS who had amenorrhea. The above mentioned studies included PCOS patients who had achieved natural pregnancy. The relationship between menstrual patterns and pregnancy outcomes in PCOS patients through IVF/ICSI has not been reported yet. This study showed that menstrual pattern is an independent factor influencing adverse pregnancy outcomes. Compared with the normal menstruation group, the amenorrhea group had a high risk for adverse pregnancy outcomes (OR: 2.039, 95% CI: 1.087–3.822).

Presently, some evidence shows that the occurrence of GDM in women who have achieved natural pregnancy may be related to the characteristics of menstruation before pregnancy. A retrospective study that included 85 patients with natural pregnancy and GDM showed that the incidence of irregular menstruation in the GDM group was significantly higher than that in the control group (24% vs. 7%; P = 0.006) (27). Another prospective cohort study of 3,600 women with natural pregnancy showed that women with long menstrual cycles (>36 days) have a higher risk of GDM than women with normal menstrual cycles (25–30 days) (OR: 1.6, 95% CI: 0.98–2.67) (28). A prospective cohort study of 578 GDM cases indicated that women with irregular menstrual cycles are at a 65% higher risk of GDM than women with regular menstrual cycles. Compared with women with a menstrual cycle between 26 and 31 days, women with a menstrual cycle usually ≥32 days are similarly at a higher risk of GDM (OR: 1.42, 95% CI: 1.15–1.75) (29). The abovementioned studies suggest that in women who have achieved natural pregnancy, irregular menstruation and prolonged menstruation (>36 days) are risk factors for GDM; however, the influence of amenorrhea on GDM and the influence of clinical outcomes in IVF/ICSI-assisted pregnancy has not been reported yet. This study shows significant differences in the GDM rates of the three groups of menstrual patterns. Menstrual pattern is an independent risk factor for GDM, and the incidence of GDM is higher in the amenorrhea group than in the normal menstrual group (OR:5.473, 95% CI: 1.189–25.178). In pregnant women with PCOS, the increase in IR caused by placental hormones has aggravated the existing IR, leading to an increased incidence of GDM. Therefore, the increased incidence of GDM in amenorrhea patients may be due to the more severe IR (30–32)

The degree of increased blood glucose in pregnant women is significantly related to the increased risk of macrosomia, sPTB, HDP, and PROM (33). The fetuses of pregnant women with GDM are three times larger than those of pregnant women with normal blood sugar levels. In this study, compared with the normal menstrual group, the incidence of macrosomia in the oligomenorrhea group and the amenorrhea group was significantly high. Multivariate logistic analysis showed that the menstrual pattern was an independent risk factor for macrosomia. Amenorrhea was higher risk than that in the normal menstrual group (OR: 4.056, 95% CI: 1.293–12.729). Macrosomia increases the risk of shoulder dystocia, clavicle fractures and brachial plexus injuries, admission to the neonatal intensive care unit and mothers’ cesarean section, postpartum hemorrhage, and vaginal lacerations (34). Similarly, pregnant women with poor blood sugar control are at increased risk of developing HDP, premature delivery, and type 2 diabetes in the future (35).

The relationship between the adverse pregnancy outcomes of PCOS patients and obesity is controversial. Our research shows that BMI is an independent risk factor for adverse pregnancy outcomes, GDM, and macrosomia. A meta-analysis indicated that the increased incidence of adverse perinatal outcomes, such as GDM, eclampsia, and preterm birth, that occurred in PCOS patients is related to BMI (36), consistent with the results of our study. However, some researchers believe that perinatal complications, such as GDM and gestational hypertension in PCOS patients, are affected by PCOS and not associated with BMI (37, 38).

In this study, the relationship between menstrual patterns among patients with PCOS and IVF/ICSI-assisted pregnancy outcomes was analyzed for the first time. Menstrual patterns were proven to be an independent risk factor for adverse pregnancy outcomes, GDM, and macrosomia. Furthermore, to reduce the impact of different protocols on pregnancy outcomes, we only included patients who received the long-acting long protocol. Simultaneously, our study had some limitations. We performed only a retrospective study, which did not consider all confounding factors. Thus, large sample, multicenter, prospective studies are still needed to confirm our findings.

In summary, our data showed that menstrual pattern is an independent risk factor for adverse pregnancy outcomes, GDM and macrosomia. The incidence of adverse pregnancy outcomes, GDM and macrosomia, in patients with amenorrhea was higher than that in the normal menstrual group and oligomenorrhea group; however, the three groups had similar clinical pregnancy rate and live birth rate. Thus, during assisted reproduction, individualized treatment should be provided according to differences in patients’ characteristics. Similarly, attention should be paid to the perinatal monitoring of patients with amenorrhea to reduce the occurrence of adverse outcomes among mothers and infants.

## Data Availability Statement

The raw data supporting the conclusions of this article will be made available by the authors, without undue reservation.

## Ethics Statement

The studies involving human participants were reviewed and approved by The First Affiliated Hospital of Zhengzhou University. Written informed consent for participation was not required for this study in accordance with the national legislation and the institutional requirements.

## Author Contributions

JZ contributed to the conception of study. TY was responsible for study designing, statistical analyses, and manuscript writing. DW contributed to revising the manuscript. YC contributed to collecting data. All authors contributed to the article and approved the submitted version.

## Funding

This study was supported by the National Natural Science Foundation of China (No. 82071649).

## Conflict of Interest

The authors declare that the research was conducted in the absence of any commercial or financial relationships that could be construed as a potential conflict of interest.

## Publisher’s Note

All claims expressed in this article are solely those of the authors and do not necessarily represent those of their affiliated organizations, or those of the publisher, the editors and the reviewers. Any product that may be evaluated in this article, or claim that may be made by its manufacturer, is not guaranteed or endorsed by the publisher.

## Supplementary Material

The Supplementary Material for this article can be found online at: https://www.frontiersin.org/articles/10.3389/fendo.2021.740377/full#supplementary-material
