# Supplementary material for: Association Between Menstrual Patterns and Adverse Pregnancy Outcomes in Patients With Polycystic Ovary Syndrome
Source: Front Endocrinol (Lausanne). 2021 Nov 18;12:740377. doi: 10.3389/fendo.2021.740377 (PMC8637325; doi:10.3389/fendo.2021.740377)
Supplement: Supplementary file 1 [file Table_1.docx]

**TABLE 1** Comparison of patients (with normal or adverse pregnancy)’ general characteristics and ovulation induction information.

| Item | normal pregnancy  (n=574) | adverse pregnancy  (n=265) | P value |
| --- | --- | --- | --- |
| menstrual patterns |  |  | 0.013 |
| regular menstruation | 10.98(63/574) | 8.30(22/265) |  |
| oligomenorrhea | 78.92(453/574) | 74.72(198/265) |  |
| amenorrhea | 10.10(58/574) | 16.98(45/265) |  |
| Age (year) | 28.49±3.42 | 28.86±3.46 | 0.144 |
| Type of infertility |  |  | 0.179 |
| Primary infertility(%) | 68.47(393/574) | 63.77(169/265) |  |
| Secondary infertility (%) | 31.53(181/574) | 36.23(96/265) |  |
| Duration of infertility (year) | 3.89±2.54 | 4.03±2.87 | 0.499 |
| BMI(kg/m2) | 24.25±3.36 | 24.77±3.32 | 0.039 |
| E2(pg/ml) | 54.70±141.97 | 69.01±194.54 | 0.234 |
| P(ng/ml) | 0.73±1.76 | 0.68±1.79 | 0.698 |
| T(ng/ml） | 0.86±4.84 | 0.90±5.53 | 0.912 |
| LH/FSH (mIU/ml) | 1.64±1.11 | 1.74±1.28 | 0.266 |
| AMH(ng/ml） | 7.35±3.61 | 7.45±3.76 | 0.716 |
| AFC (n) | 22.42±4.30 | 21.89±5.19 | 0.152 |
| Starting dose of Gn(IU) | 108.34±17.84 | 110.70±18.79 | 0.081 |
| Length of stimulation (d) | 14.41±2.60 | 14.57±2.56 | 0.403 |
| Total dos age of Gn used (IU) | 2153.83±882.70 | 2273.54±868.52 | 0.067 |
| hCG injection day |  |  |  |
| Endometrial thickness(mm) | 12.73±2.33 | 12.21±2.33 | 0.003 |
| E2 (pg/ml) | 3472.68±1826.01 | 3216.60±1553.23 | 0.050 |
| LH (mIU/ml) | 0.78±1.05 | 0.71±0.77 | 0.309 |
| P (ng/ml) | 0.74±0.48 | 0.73±0.46 | 0.905 |
| No. of oocytes retrieved(n) | 16.05±5.54 | 15.33±5.79 | 0.088 |

Note:Continuous data: mean±SD. Categorical data: % (n/N).

**TABLE 2** Logistic regression assessment of adverse pregnancy outcomes.

| Item | OR(95%CI) | P | AORa(95%CI) | P^a^ |
| --- | --- | --- | --- | --- |
| menstrual patterns |  | 0.014 |  | 0.042 |
| regular menstruation | Reference | - | Reference | - |
| oligomenorrhea | 1.252(0.749,2.091) | 0.391 | 1.246(0.742,2.091) | 0.405 |
| amenorrhea | 2.222(1.193,4.139) | 0.012 | 2.039(1.087,3.822) | 0.026 |
| BMI(kg/m^2^) | 1.047(1.002,1.094) | 0.039 | 1.046(1.001,1.093) | 0.043 |
| hCG injection day  Endometrial thickness(mm) | 0.907(0.851,0.967) | 0.003 | 0.91(0.853,0.971) | 0.004 |

Note: Reference, This variable functions as an indicator; ^a^OR, ^a^P, adjusted by the variables which were statistically significant in the univariate logistic analysis; Menstrual patterns of adverse pregnancy adjusted by BMI and hCG injection day endometrial thickness.

**TABLE 3** Comparison of patients (with or without GDM)’ general characteristics and ovulation induction information.

| Item | No GDM  (n=790) | GDM  (n=49) | P value |
| --- | --- | --- | --- |
| menstrual patterns |  |  | 0.022 |
| regular menstruation | 10.51(83/790) | 4.08(2/49) |  |
| oligomenorrhea | 77.97(616/790) | 71.43(35/49) |  |
| amenorrhea | 12.28(97/790) | 24.49(12/49) |  |
| Age (year) | 28.54±3.41 | 29.71±3.62 | 0.020 |
| Type of infertility |  |  | 0.797 |
| Primary infertility(%) | 67.09(530/790) | 65.31(32/49) |  |
| Secondary infertility (%) | 32.91(260/790) | 34.69(17/49) |  |
| Duration of infertility (year) | 3.94±2.62 | 3.77±3.10 | 0.659 |
| BMI(kg/m2) | 24.34±3.37 | 25.90±2.87 | 0.006 |
| E2(pg/ml) | 57.83±148.85 | 81.34±289.67 | 0.320 |
| P(ng/ml) | 0.74±1.81 | 0.34±0.27 | 0.138 |
| T(ng/ml） | 0.90±5.22 | 0.44±0.37 | 0.537 |
| LH/FSH (mIU/ml) | 1.69±1.17 | 1.41±0.99 | 0.102 |
| AMH(ng/ml） | 7.41±3.69 | 6.95±3.08 | 0.394 |
| AFC (n) | 22.24±4.62 | 22.41±4.39 | 0.808 |
| Starting dose of Gn(IU) | 108.80±18.23 | 113.78±16.58 | 0.063 |
| Length of stimulation (d) | 14.45±2.59 | 14.69±2.62 | 0.518 |
| Total dos age of Gn used (IU) | 2175.13±868.81 | 2457.91±1009.76 | 0.061 |
| hCG injection day |  |  |  |
| Endometrial thickness(mm) | 12.58±2.34 | 12.27±2.40 | 0.356 |
| E2(pg/ml) | 3418.22±1764.15 | 2968.42±1404.37 | 0.080 |
| LH (mIU/ml) | 0.77±0.98 | 0.61±0.64 | 0.277 |
| P (ng/ml) | 0.74±0.48 | 0.69±0.37 | 0.497 |
| No. of oocytes retrieved(n) | 15.79±5.53 | 16.37±6.99 | 0.483 |

Note:Continuous data: mean±SD. Categorical data: % (n/N).

**TABLE 4** Logistic regression assessment of GDM.

|  | OR(95%CI) | P | AORa(95%CI) | APa |
| --- | --- | --- | --- | --- |
| menstrual patterns |  | 0.020 |  | 0.048 |
| regular menstruation | Reference | - | Reference | - |
| oligomenorrhea | 2.358(0.557,9.985) | 0.244 | 2.462(0.579,10.470) | 0.222 |
| amenorrhea | 5.473(1.189,25.178) | 0.029 | 5.023(1.083,23.289) | 0.039 |
| Age (year) | 1.100(1.015,1.192) | 0.020 | 1.086(1.003,1.177) | 0.043 |
| BMI(kg/m^2^) | 1.128(1.035,1.230) | 0.006 | 1.117(1.022,1.220) | 0.014 |

Note: Reference, This variable functions as an indicator; ^a^OR, ^a^P, adjusted by the variables which were statistically significant in the univariate logistic analysis; Menstrual patterns of GDM adjusted by Age and BMI.

**TABLE 5** Comparison of patients (with or without macrosomia)’ general characteristics and ovulation induction information.

| Item | No macrosomia  (n=664) | Macrosomia  (n=84) | P value |
| --- | --- | --- | --- |
| menstrual patterns |  |  | 0.026 |
| regular menstruation | 11.18(72/644) | 4.76(4/84) |  |
| oligomenorrhea | 80.90(521/644) | 76.19(64/84) |  |
| amenorrhea | 11.02(71/644) | 19.05(16/84) |  |
| Age (year) | 28.56±3.42 | 28.55±3.29 | 0.981 |
| Type of infertility |  |  | 0.344 |
| Primary infertility(%) | 70.34(453/644) | 63.10(53/84) |  |
| Secondary infertility (%) | 32.76(211/644) | 36.90(31/84) |  |
| Duration of infertility (year) | 3.87±2.40 | 4.13±3.30 | 0.391 |
| BMI(kg/m2) | 24.20±3.38 | 25.61±3.08 | <0.001 |
| E2(pg/ml) | 58.71±158.43 | 77.57±241.14 | 0.341 |
| P(ng/ml) | 0.72±1.84 | 0.71±1.38 | 0.947 |
| T(ng/ml） | 0.83±4.80 | 0.78±3.46 | 0.92 |
| LH/FSH (mIU/ml) | 1.65±1.19 | 1.58±0.88 | 0.619 |
| AMH(ng/ml） | 7.26±3.60 | 7.53±3.61 | 0.521 |
| AFC (n) | 22.06±4.83 | 23.48±1.86 | 0.008 |
| Starting dose of Gn(IU) | 108.28±18.37 | 115.18±18.42 | 0.001 |
| Length of stimulation (d) | 14.40±2.54 | 14.52±2.84 | 0.672 |
| Total dos age of Gn used (IU) | 2158.74±891.83 | 2322.92±870.65 | 0.111 |
| hCG injection day |  |  |  |
| Endometrial thickness(mm) | 12.62±2.38 | 12.37±2.00 | 0.350 |
| E2 (pg/ml) | 3421.55±1821.93 | 3202.80±1318.52 | 0.290 |
| LH (mIU/ml) | 0.76±0.99 | 0.83±1.09 | 0.524 |
| P (ng/ml) | 0.73±0.46 | 0.71±0.49 | 0.799 |
| No. of oocytes retrieved(n) | 15.55±5.44 | 18.35±6.09 | <0.001 |
| No.of live babies delivered |  |  | <0.001 |
| Singletons rate (%) | 79.19(510/644) | 100.00(84/84) |  |
| Multiples rate (%) | 23.91(154/644) | 0.00(0/84) |  |

Note:Continuous data: mean±SD. Categorical data: % (n/N).

**TABLE 6** Logistic regression assessment of macrosomia

| Item | OR(95%CI) | P | AORa(95%CI) | P^a^ |
| --- | --- | --- | --- | --- |
| menstrual patterns |  | 0.032 |  | 0.028 |
| regular menstruation | Reference | - | Reference | - |
| oligomenorrhea | 2.211(0.782,6.254) | 0.135 | 2.992(1.036,8.647) | 0.043 |
| amenorrhea | 4.056(1.293,12.729) | 0.016 | 4.918(1.516,15.954) | 0.008 |
| BMI(kg/m2) | 1.133(1.058,1.213) | <0.001 | 1.021(1.004,1.037) | 0.014 |
| AFC (n) | 1.157(1.025,1.305) | 0.018 | 1.196(1.044,1.37) | 0.010 |
| Starting dose of Gn(IU) | 1.017(1.006,1.028) | 0.002 | 1.041(0.948,1.144) | 0.400 |
| No. of oocytes retrieved(n) | 1.089(1.047,1.133) | <0.001 | 1.057(1.014,1.101) | 0.009 |
| Multiples rate | / | 0.995 | / | 0.995 |

Note: Reference, This variable functions as an indicator; ^a^OR, ^a^P, adjusted by the variables which were statistically significant in the univariate logistic analysis; Menstrual patterns of macrosomia adjusted by BMI, AFC, Starting dose of Gn, No. of oocytes retrieved and Multiples rate; /,because of the sample size, the OR value cannot be displayed.
